# Potential Neuroprotective Treatment of Stroke: Targeting Excitotoxicity, Oxidative Stress, and Inflammation

**DOI:** 10.3389/fnins.2019.01036

**Published:** 2019-09-27

**Authors:** Qianwen Yang, Qianyi Huang, Zhiping Hu, Xiangqi Tang

**Affiliations:** Department of Neurology, The Second Xiangya Hospital of Central South University, Changsha, China

**Keywords:** excitotoxicity, inflammation, neuroprotective, potential treatment, ROS, stroke

## Abstract

Stroke is a major cause of death and adult disability. However, therapeutic options remain limited. Numerous pathways underlie acute responses of brain tissue to stroke. Early events following ischemic damage include reactive oxygen species (ROS)-mediated oxidative stress and glutamate-induced excitotoxicity, both of which contribute to rapid cell death within the infarct core. A subsequent cascade of inflammatory events escalates damage progression. This review explores potential neuroprotective strategies for targeting key steps in the cascade of ischemia–reperfusion (I/R) injury. NADPH oxidase (NOX) inhibitors and several drugs currently approved by the U.S. Food and Drug Administration including glucose-lowering agents, antibiotics, and immunomodulators, have shown promise in the treatment of stroke in both animal experiments and clinical trials. Ischemic conditioning, a phenomenon by which one or more cycles of a short period of sublethal ischemia to an organ or tissue protects against subsequent ischemic events in another organ, may be another potential neuroprotective strategy for the treatment of stroke by targeting key steps in the I/R injury cascade.

## Introduction

Although stroke is a major cause of death and adult disability, therapeutic options remain limited. The development of new treatments including potential pharmaceutical agents is therefore of great importance. The acute responses of brain tissue to cerebral ischemia are complex. First, oxidative stress, which plays an essential role in the pathogenesis of cerebral ischemia–reperfusion (I/R) injury (Zalba et al., 2007; Carbone et al., 2015), is caused by increased reactive oxygen species (ROS) production and decreased activity levels of scavenger enzymes and protective antioxidants (De Silva and Miller, 2016; Grochowski et al., 2017). Second, glutamate, the most abundant excitatory neurotransmitter, acts as a potent neurotoxin under pathological conditions. Increased extracellular glutamate levels play an essential role in ischemia-mediated cytotoxicity through *N*-methyl-D-aspartate (NMDA) and α-amino-3-hydroxy-5-methyl-4-isoxazolepropionic acid (AMPA) ionotropic glutamate receptors (Chuang et al., 2011; Vaarmann et al., 2013; Khanna et al., 2015; Yoo et al., 2017). Third, minutes to hours after cerebral ischemia onset, a series of inflammatory events are triggered following the activation of resident cells including microglia. Several signals contribute to the two main activation phenotypes: classically activated (M1) and alternatively activated (M2) (Kim et al., 2015; Bonaventura et al., 2016; Fu and Yan, 2018). Microglia are sensitive to signaling through receptors such as toll-like receptors (TLRs) and peroxisome proliferator-activated receptor-γ (PPAR-γ) (Kim et al., 2015). Primary signals that upregulate inflammatory mediators include damage-associated molecular patterns (DAMPs) (Macrez et al., 2011; Bonaventura et al., 2016). Other signals are hyaluronan and pathogen-associated molecular patterns (PAMPs). Many DAMPs and PAMPS are sensed by TLRs (Macrez et al., 2011). The M1 phenotype promotes the release of inflammatory mediators such as nitric oxide and ROS. This leads to increased cell death and blood–brain barrier dysfunction, triggering the release of chemokines, activating matrix metalloproteinase (MMP)-9, and upregulating adhesion molecules. The M2 phenotype is activated by anti-inflammatory cytokines such as interleukin-4, which may inhibit inflammation and promote tissue repair and wound healing (Macrez et al., 2011; Kim et al., 2015; Bonaventura et al., 2016). The development of novel neuroprotective strategies to target key steps in this cascade may represent promising therapeutic options. Therefore, this review explores potential neuroprotective strategies for halting the cascade of I/R injury. These neuroprotective strategies include NADPH oxidase (NOX) inhibitors and drugs currently approved by the Food and Drug Administration to treat other diseases but show promise as new drugs for the treatment of stroke in animal experiments and clinical trials. Ischemic conditioning may be another neuroprotective strategy for stroke.

## Potential Interventions

### NOX Inhibitor

Inhibition of ROS production may be a viable strategy for the treatment of stroke. Cyclooxygenases and mitochondria generate ROS as a byproduct of their enzymatic activities; in contrast, ROS generation is the main function of NOXs. The NOX family includes the following subtypes: NOX1, NOX2, NOX3, and NOX4 (Chen et al., 2011b; De Silva and Miller, 2016; Grochowski et al., 2017; Carvalho and Moreira, 2018). NOX2 and NOX4 activity makes a major contribution to oxidative stress following stroke (Park et al., 2007; Chen et al., 2011a; Casas et al., 2017; Carvalho and Moreira, 2018).

Targeting NOXs may be an effective strategy for the treatment of stroke (Yao et al., 2017). The most common NOX inhibitor is apocynin, a naturally occurring methoxy-substituted catechol. In experimental stroke, it could decrease infarct volume by reducing levels of apoptosis and inhibiting oxidative stress. Apocynin also induce adaptation to ischemic inflammation, but its therapeutic window is narrow (Genovese et al., 2011). Diphenyleneiodonium is another common NOX inhibitor, it attenuates blood–brain barrier damage and improves neurological outcome after focal cerebral ischemia in animal models. However, the effects of most NOXs are non-specific (Nagel et al., 2007). Gp91ds-tat may be the most specific NOX inhibitor, it includes a conserved sequence of Nox2/gp91phox linked to a 9-amino-acid peptide from the human immunodeficiency virus coat, which allows the peptide to penetrate cells. However, because it is a peptide, its utility is limited by low oral bioavailability (De Silva and Miller, 2016). Ebselen (2-phenyl-1,2-benzisothiazol-3 (2H)-one), a selenium-containing organic antioxidant with low toxicity, is another promising NOX inhibitor that may be beneficial to combat excessive apoptosis in patients with stroke, but it is associated with side effects (Yamagata et al., 2008; Kim et al., 2017). Spirulina is a nutritional supplement rich in proteins and antioxidants that has been shown to attenuate middle cerebral artery occlusion (MCAO)-induced focal cerebral ischemia in rodents; it acts cooperatively at different stages of free radical generation to protect the neurons from focal cerebral I/R injury (Thaakur and Sravanthi, 2010).

NADPH oxidase inhibitors are a promising treatment, but they have low specificity and many side effects. At present, this regards animal experiments of the NOX inhibitor when used for strokes, and more research is needed in the future.

### Hypoglycemic Drugs

Peroxisome proliferator-activated receptors (PPARs) are ligand-activated transcription factors of the nuclear hormone receptor superfamily. PPAR-γ is one of the isoforms, and agonists of PPAR-γ may be beneficial to I/R injury. The thiazolidinediones (TZDs) include rosiglitazone and pioglitazone; these synthetic agonists of PPAR-γ are widely involved in glycemic control and are approved as glucose-lowering agents. They also represent promising drug therapy for ischemic stroke. These drugs exert neuroprotection in normotensive, normoglycemic, or hypertensive type-2 diabetic rodents. TZD treatment may decrease infarct volumes and neurological deficits in rodents. The major mechanism is inhibition of oxidative stress and excessive inflammatory responses (Collino et al., 2006; Tureyen et al., 2007). In 2010, a meta-analysis of experimental studies involving TZD treatment in rodent models of cerebral ischemia showed they may be beneficial for ischemic stroke (White and Murphy, 2010). In 2014, a systematic review evaluated the effectiveness and safety of PPAR-γ agonists in the secondary prevention of stroke. Three studies evaluated pioglitazone and one assessed rosiglitazone. Three studies included participants who had no history of diabetes, and one study only included participants with diabetes. The results showed that PPAR-γ agonists were effective; they reduced recurrent strokes and other vascular events, improved insulin sensitivity, and stabilized carotid plaques (Liu and Wang, 2017).

Other common drugs for treatment of type 2 diabetes, such as metformin, a biguanide drug, may also benefit stroke patients (Deng et al., 2016; Mima et al., 2016; Zhou et al., 2016). In an experimental study, pretreatment with 10 mg/kg metformin for 7 days was neuroprotective; it improved neurological scores and decreased cell apoptosis in mice with permanent MCAO. However, after cerebral ischemia onset, metformin may induce opposite effects to those seen with pretreatment. These phenomena require further research (Deng et al., 2016; Arbelaez-Quintero and Palacios, 2017; Karimipour et al., 2018). In addition to being beneficial to mice with MCAO, in another experimental study, metformin may also be of potential utility for the treatment of intracerebral hemorrhage (ICH). It protected rats from neurological deficits induced by ICH, possibly through inhibiting apoptosis, oxidative stress, and neuroinflammation (Qi et al., 2017).

Sulfonylureas are hypoglycemic drugs that are commonly used in the treatment of type 2 diabetes mellitus (T2DM), including ATP-sensitive potassium channel and/or sulfonylurea receptor 1 (SUR1) receptor blockers. Several studies reported that they are also beneficial for acute ischemic stroke (Kunte et al., 2007; Wali et al., 2012; Sheth et al., 2016). The sulfonylurea glibenclamide showed potential therapeutic utility in I/R brain injury via modulating oxidative stress and inflammatory mediators (Abdallah et al., 2011). Another group reported that glibenclamide mainly targeted SUR1-regulated non-selective cation channel-ATP channel-mediated cerebral edema after ischemic stroke (Simard et al., 2006).

Many hypoglycemic drugs are considered useful in the treatment of stroke, but the results are still preliminary. We should interpret the conclusions of some systematic reviews with caution because the number of included studies may be small, and the quality is relatively low.

### Minocycline

Minocycline, a broad-spectrum second-generation semisynthetic antibiotic and tetracycline derivative, has been used to treat a variety of infectious diseases such as meningitis, respiratory infections, acne vulgaris, shigellosis, conjunctivitis, psittacosis, and chlamydia. It also has been used to treat rheumatoid arthritis (Fagan et al., 2011).

In recent years, neuroprotective properties of minocycline have been reported. Numerous studies have demonstrated the efficacy of minocycline in animal models and clinical trials of stroke. Its mechanisms of action may be anti-inflammatory, inhibition of MMPs (Elewa et al., 2006). Most findings were in preclinical experimental studies in animal models (Liu et al., 2007), but it is also being assessed in clinical trials.

Two clinical studies found neuroprotective effects of oral minocycline on ischemic stroke. In both studies, patients all received oral minocycline 200 mg/day for 5 days and were compared with subjects who received placebo; clinical assessment was performed using the National Institutes of Health Stroke Scale (NIHSS) score, modified Rankin Scale (mRS), and Barthel index (BI). Results showed significant neurological improvement on days 30 and 90. In addition, deaths, myocardial infarctions, recurrent strokes, and hemorrhagic transformations were not different between the two groups (Lampl et al., 2007; Padma Srivastava et al., 2012). These results were consistent with an open-label evaluator-blinded clinical study in which patients also received 200 mg/day oral minocycline for 5 days. However, the results were sex dependent; female patients showed no significant clinical improvement compared with males (Amiri-Nikpour et al., 2015). The reason for this difference is unclear.

The efficacy of minocycline in acute ICH is uncertain. Two different clinical trials reported no significant effects compared to placebo. In one of these studies, intravenous minocycline was administered at a dose of 10 mg/kg once a day for 5 days total (Chang et al., 2017). In the other, patients received 400 mg of intravenous minocycline, followed by 400 mg oral minocycline daily for 4 days (Fouda et al., 2017). Another pilot study administered 100 mg of minocycline intravenously up to five doses in patients with ischemic or hemorrhagic stroke and found that it was not effective (Kohler et al., 2013).

The discrepant results between clinical studies may be attributable to stroke type, sex differences, drug doses, sample sizes, and the route of minocycline administration. In 2018, a systematic review and meta-analysis of randomized clinical trials (RCTs) about minocycline for acute stroke treatment identified seven RCTs including a total of 426 patients. The authors concluded that minocycline demonstrated efficacy, especially in patients with acute ischemic stroke. The efficacy and safety of minocycline for ICH patients need further exploration. In addition, in a subgroup analysis of RCTs comparing the route of minocycline administration, the oral route was significantly associated with a higher likelihood of 3 month functional independence and clinical improvements for mean 3 month NIHSS score and mean 3 month BI, compared with the intravenous route (Malhotra et al., 2018).

Minocycline has been widely studied in animal and clinical trials of stroke, and some encouraging results have been achieved. Nonetheless, the application of minocycline in stroke is still limited; further large-scale studies are required to clarify sex-, dose- and route-dependent effects.

### Fingolimod

Fingolimod is an oral sphingosine-1-phosphate receptor analog used to treat relapsing-remitting multiple sclerosis (MS). It decreases lymphocyte entry from secondary lymphoid tissues, reduces central nervous system inflammation, and may be a promising drug for stroke (Tsai and Han, 2016). Following consistent benefits demonstrated in stroke animal models, some research groups have started to assess fingolimod in clinical trials. To date, the results of four clinical studies have been published (Fu et al., 2014a, b; Zhu et al., 2015; Tian et al., 2018).

Three clinical studies reported neuroprotective effects of oral fingolimod on ischemic stroke. In 2014, an open-label, evaluator-blinded, non-randomized study was published. The authors examined the effect of standard management plus fingolimod on ischemic stroke compared to standard management alone. The control (*n* = 11) and the study (*n* = 11) groups had comparable baseline characteristics. Study group patients received oral fingolimod (0.5 mg/day for 3 consecutive days), and clinical assessment was performed using NIHSS score, mRS, and modified BI. The 11 fingolimod recipients had lower circulating lymphocyte counts, milder neurological deficits, better recovery of neurological functions, more restrained enlargement of lesion size, and lower microvascular permeability. There were no serious drug-related events (Fu et al., 2014b). In 2015, a multi-center study was conducted to assess the efficacy of fingolimod in conjunction with alteplase. A total of 47 patients were randomly assigned to receive alteplase alone (*n* = 25) or with fingolimod (*n* = 22; oral fingolimod 0.5 mg daily for 3 consecutive days within 4.5 h of the onset of ischemic stroke). Compared with patients who received alteplase alone, patients who also received fingolimod exhibited lower circulating lymphocytes, smaller lesion volumes, less hemorrhage, attenuated neurologic deficits on the NIHSS, restrained lesion growth, and better recovery of neurological functions. No serious adverse events were recorded in any patients (Zhu et al., 2015).

In 2018, a prospective, randomized, open-label, blinded endpoint clinical trial was conducted to determine the efficacy of coadministration of fingolimod with alteplase in acute ischemic stroke patients in a delayed time window. Patients with internal carotid or middle cerebral artery proximal occlusion within 4.5–6 h of symptom onset were enrolled and randomly assigned to receive alteplase alone or with fingolimod; each group included 23 patients. Fingolimod may augment the efficacy of alteplase in the 4.5–6 h time window in proximal cerebral arterial occlusion patients and help salvage penumbral tissue by promoting both anterograde reperfusion and retrograde collateral flow (Tian et al., 2018). These studies suggested that combination therapy of fingolimod and alteplase was well tolerated, attenuated reperfusion injury, and improved clinical outcomes. It has also been shown to be effective in the extended time window of 4.5–6 h.

A two-arm proof-of-concept study investigated the safety and effectiveness of fingolimod in ICH patients based on the hypothesis that modulation of brain inflammation reduces edema. It included 23 patients with primary supratentorial ICH with a hematoma volume of 5–30 mL. All patients received standard management alone or combined with fingolimod (0.5 mg, orally for 3 consecutive days). Treatment was initiated within 72 h after symptom onset. The administration of oral fingolimod was safe, reduced ICH, attenuated neurologic deficits, and promoted recovery (Fu et al., 2014b).

### Dimethyl Fumarate

Dimethyl fumarate (DMF) is another drug used to treat MS. Monomethyl–fumarate, a metabolite of DMF, modulates inflammatory responses, stimulates antioxidant pathways, and may also be effective for treating cerebral I/R injury (Yao et al., 2016). These effects may be achieved through enhancing nuclear factor erythroid-2-related factor two signaling, an endogenous survival pathway that exerts antioxidant and anti-inflammatory effects in cerebral I/R injury and ICH (Linker et al., 2011; Zhao et al., 2015; Fowler et al., 2018). DMF has been shown to reduce neurological deficits, infarct volume, brain edema, and cell death (Kunze et al., 2015; Lin et al., 2016; Yao et al., 2016; Clausen et al., 2017).

Fingolimod and DMF are immunomodulators and promising drugs in the treatment of stroke. However, most studies have been performed in animal models, and more clinical trials are needed.

### Ischemic Conditioning

Ischemic conditioning is a phenomenon by which one or more cycles of a short period of sublethal ischemia to an organ or tissue protects against subsequent ischemic events in another organ (Hahn et al., 2011). The protective conditioning cycles are applied to an organ or tissue remote from the target organs and may be an effective strategy against stroke (Zhao W. et al., 2018). Remote limb ischemic conditioning (RLIC) involves repetitive inflation and deflation of a blood pressure cuff on the limb; it produces repetitive, transient ischemia and protects against subsequent ischemic in brain (Al Kasab et al., 2016). This approach can be divided into three types: preconditioning, perconditioning, and postconditioning (Wang et al., 2015). Preclinical studies have demonstrated its potential benefit for ischemic stroke in animal models (Ren et al., 2015). The protective mechanisms of RLIC may involve the following aspects: anti-oxidant, anti-cell death, anti-inflammation and anti-edema, but the exact mechanisms remain to be elucidated (Chen et al., 2018).

Many clinical studies have shown RLIC to be a well-tolerated and effective strategy for stroke patients suffering from or at risk of brain I/R injury (Mi et al., 2016; England et al., 2017; Wang et al., 2017). However, some other studies demonstrated the neutral or contrary results. In 2018, a systematic review and meta-analysis of RCTs was published. The authors included three trials that analyzed the effects of remote ischemic conditioning (RIC) on ischemic stroke prevention and four trials that analyzed the effects of RIC on ischemic stroke treatment. The results showed that RIC decreased the risk of recurrent stroke in people with intracerebral artery stenosis and decreased stroke severity in people undergoing carotid stenting, but it increased death or dependence in people with acute ischemic stroke who were undergoing intravenous thrombolysis (Zhao W. et al., 2018). However, the interpretation of these results is limited by the small sample sizes and low quality of evidence. Another meta-analysis of RCTs published in 2018 suggested that remote ischemic postconditioning might offer cerebral protection for stroke patients suffering from or at risk of brain I/R injury and that there were no side effects (Zhao J.J. et al., 2018). However, most previous studies focused on patients younger than 80 years of age; there are few investigations about the safety and effectiveness of RLIC on stroke prevention and treatment in octo- and nonagenarians.

As it is non-invasive and easy to apply, RLIC is a promising treatment for stroke. Although clinical trials have described some positive outcomes, there is still controversy about the effectiveness and safety of RLIC for stroke prevention and treatment, especially in octo- and nonagenarians. In addition, the optimal duration and number of I/R cycles have not been established. Additional large-scale studies needed to answer these questions.

## Conclusion

Therapeutic options for stroke are limited. This review highlights potential novel interventions that target key steps in the I/R injury cascade. NOX inhibitors; drugs such as rosiglitazone, pioglitazone, metformin, sulfonylureas, minocycline, fingolimod, and dimethyl fumarate; and novel approaches such as ischemic conditioning are all potential interventions for stroke (as shown in Table 1). Among these, minocycline and ischemic conditioning are especially promising. Although some encouraging results have been published, there are still many shortcomings. Future studies should include more RCTs to confirm the efficacy of these treatments.

**TABLE 1:** Mechanisms of neuroprotective treatment.

**Treatment**	**Mechanisms**
Apocynin, diphenyleneiodonium, Gp91ds-tat, ebselen, spirulina	Inhibit NOX
Rosiglitazone and pioglitazone	Inhibits oxidative stress and the inflammatory response
Metformin	Inhibits apoptosis, oxidative stress, and neuroinflammation
Glibenclamide	Modulate oxidative stress and inflammatory mediators
Minocycline	Anti-inflammatory, MMP inhibition, and anti-apoptotic
Fingolimod	Anti-inflammatory
Dimethyl fumarate	Modulates inflammatory responses and stimulates antioxidant pathways
Ischemic conditioning	Anti-oxidant, anti-cell death, anti-inflammation, anti-edema

## Author Contributions

QY primarily wrote the manuscript. QH contributed to the acquisition or analysis of literature for the work. ZH contributed to editing the review. XT supervised the work, helped with summarizing the manuscript, and contributed to the review revision. All authors read and approved the final manuscript.

## Conflict of Interest

The authors declare that the research was conducted in the absence of any commercial or financial relationships that could be construed as a potential conflict of interest.

## Abbreviations

AMPA: α-amino -3-hydroxy-5-methyl-4-isoxazolepropionic acid
BI: barthel index
DAMPs: damage-associated molecular patterns
DMF: dimethyl fumarate
ICH: intracerebral hemorrhage
I/R: ischemia–reperfusion
mRS: modified Rankin scale
MS: multiple sclerosis
NIHSS: National Institutes of Health Stroke Scale
NMDA: *N*-methyl -D-aspartate
NOX: NADPH oxidase
PAMPs: pathogen-associated molecular patterns
PPAR γ: peroxisome proliferator-activated receptor- γ
RCTs: randomized clinical trials
RLIC: remote limb ischemic conditioning
RIC: remote ischemic conditioning
ROS: reactive oxygen species
SUR1: sulfonylurea receptor 1
TLRs: toll-like receptors
TZD: thiazolidinedione
T2DM: type 2 diabetes mellitus.
